# The use of human sewage screening for community surveillance of hepatitis E virus in the UK

**DOI:** 10.1002/jmv.24403

**Published:** 2015-11-04

**Authors:** Donald B. Smith, Julius O. Paddy, Peter Simmonds

**Affiliations:** ^1^ CIIEAshworth LaboratoriesKing's BuildingsUniversity of EdinburghEdinburghScotlandUnited Kingdom; ^2^ Roslin InstituteEaster BushUniversity of EdinburghEdinburghScotlandUnited Kingdom

**Keywords:** epidemiology, variation, detection

## Abstract

Hepatitis E virus sequences were detected by RT‐PCR in 14/15 (93%) of untreated sewage samples from Edinburgh, Scotland, UK. Phylogenetic analysis of amplicons at limiting dilution revealed the co‐circulation of multiple variants of HEV‐3, with a pattern of diversity matching that observed in a local cohort of HEV‐infected hepatitis patients. ***J. Med. Virol. 88:915–918, 2016*.** © 2015 The Authors. *Journal of Medical Virology* Published by Wiley Periodicals, Inc.

Hepatitis E virus (HEV) is a single stranded positive‐sense RNA virus (Family: *Hepeviridae*, Genus: *Orthohepevirus*, Species: *Orthohepevirus A*) that is increasingly recognized as a significant cause of hepatitis in the developed world. The first report that HEV could be detected in sewage was published more than 20 years ago [Jothikumar et al., 1993]. Subsequent reports describe the frequent detection of HEV in sewage from France (25%), Switzerland (32%), India (56%), and Spain (32–43%) although lower figures are described in other studies (Table I). The frequency of detection can vary with season, being 14% in the winter and 50% in the summer in Switzerland [Masclaux et al., 2013], and also over time, varying from 3% to 84% in Barcelona [Pina et al., 1998; Clemente‐Casares et al., 2003]. Although several of these studies included nucleotide sequence analysis of amplicons, these comprised single or small numbers of sequences and frequently utilized genomic regions that were too short (<150 nucleotides) for reliable phylogenetic analysis. In consequence, there is at present no adequate description of the genetic diversity of HEV in sewage samples, nor on the potential value of sewage surveillance in documenting the community circulation of different HEV genotypes and strains.

**Table I:** HEV Detection in Urban Sewage and Waste Water

				Number of isolates	
Country (reference)	Year	Positive/total samples (%)	Sequence length (Region)	HEV‐1	HEV‐3
Spain [Pina et al., 1998]	1994–1998	1/37 (3)	5,800 nt	1	
Spain [Clemente‐Casares et al., 2009]	2000–2007	29/91 (32)	101nt (ORF2)	[(5]*	[15]
Spain [Clemente‐Casares et al., 2003]	1994–2002	20/46 (43)	123nt (ORF2)		[6]
France [Clemente‐Casares et al., 2003]		1/4 (25)	123nt (ORF2)		[1]
Greece [Clemente‐Casares et al., 2003]	1999	0/5			
Sweden [Clemente‐Casares et al., 2003]	1997	0/4			
Switzerland [Masclaux et al., 2013]	2010–2012	40/124 (32)	221nt (ORF1)	1	40^**^
Norway [Myrmel et al., 2015]	2008–2009	8/102 (8)	98nt (ORF2)	[1]	[3]
Italy [La Rosa et al., 2010]	2008–2009	19/118 (16)	127nt (ORF1)	[18]	[1]
Greece [Kokkinos et al., 2011]	2007–2009	0/48			
UK (this study)	2014–2015	14/15 (93)	302nt (ORF2)		60
Tunisia [Béji‐Hamza et al., 2014]	2007	3/150 (2)	132nt (ORF1)	[1]	[2]
Egypt [Kamel et al., 2011]	2006–2007	0/76			
India [Vivek et al., 2013]	2009–2010	80/144 (56)	325nt (ORF1)	8	
Argentina [Martínez Wassaf et al., 2014]	2007–2011	3/48 (6)	280nt (ORF2)		3
USA [Clemente‐Casares et al., 2003]	1999	1/5 (20)	123nt (ORF2)		[1]

^**^ No GenBank accession numbers and/or phylogenetic tree presented.

[]*Phylogenetic analysis based on sequences of <150 nucleotides.

The prevalence and diversity of HEV in sewage was investigated by screening pre‐treatment sewage samples obtained from Seafield Waste Water Treatment Works, Edinburgh, Scotland at 2‐week intervals from March 26, 2014–September 10, 2014 as well as on December 24, 2014 and January 7, 2015. This sewage works receives domestic sewage from the entire city of Edinburgh (population 500,000) but does not receive effluent from pig farms or other agricultural run‐off. After removal of solid matter by centrifugation, 20 ml samples were passed through a 0.45 µM filter before concentrating (×20) using a Centriplus YM‐50 spin column (Millipore 4310) and then (×10) with an Amicon filter (UFC5000324). Virus nucleic acid was extracted using the QiaAmp viral RNA kit and HEV RNA detected at limiting dilution by RT‐PCR using the Access kit (Promega) followed by nested PCR as described previously [Smith et al., 2013]. Up to 2 μl in aggregate of nucleic was tested from each sample, equivalent to 400 μl of filtered sewage. This protocol was capable of detecting 11.5 IU of the QCMID subtype 3f standard and 230 IU of the QCMID subtype 3c standard. Nucleotide sequences (ORF2 positions 6069‐6370 relative to AF082843) were deducted, aligned, and subjected to phylogenetic analysis using MEGA6, as described previously [Smith et al., 2013]. Phylogenetic relationships between complete genome reference sequences in this region were congruent with those displayed by complete genome sequences. The GenBank Accession numbers of the nucleotide sequences obtained in this study are KT230687‐KT230747.

All but one of the pre‐treatment sewage samples (14/15, 93%) were RT‐PCR positive for HEV. Phylogenetic analysis of 2‐21 HEV sequences obtained from each of the RT‐PCR positive samples at dilutions at which less than half of replicates were PCR positive revealed that all sequences were genotype HEV‐3, with the majority grouping with a subtype 3c reference sequence (Fig. 1). Other sewage‐derived sequences grouped with the subtype 3a reference sequences, while the remainder grouped with the subtype 3e and 3f reference sequences or grouped separately from any of the reference sequences. This distribution of variants within HEV‐3 is broadly similar to that observed amongst previously obtained sequences from HEV positive hepatitis patients at the Edinburgh Royal Infirmary (GenBank accession numbers KP835485‐KP835511, open circles), and to that observed in a larger study of hepatitis patients from England and Wales [Ijaz et al., 2014] but differs from that observed in UK pigs [Grierson et al., 2015].

**Figure 1:**
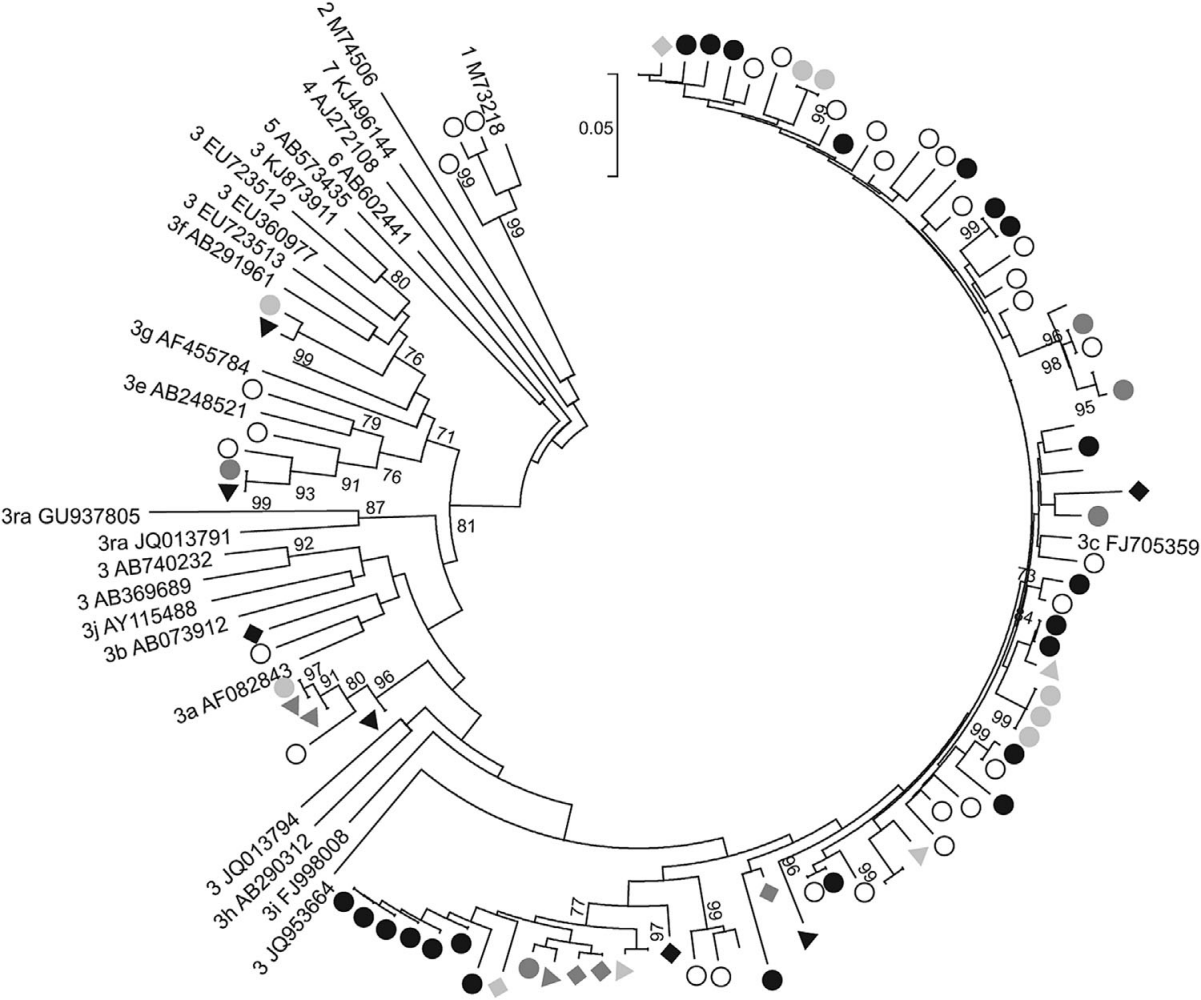
Phylogenetic analysis of HEV ORF2 sequences from Edinburgh sewage. A neighbor joining tree of maximum composite likelihood distances was produced using sequences from a 302 nucleotide region of ORF2. Sequences from different batches of sewage are indicated by solid symbols of different types while sequences from Edinburgh hepatitis patients are labelled with open circles. Branches corresponding to samples where only two independent sequences were obtained are unlabelled. Reference sequences are indicated by genotype and GenBank accession number. Branches supported by >70% of bootstrap replicates are indicated.

Multiple HEV variants were present in several sewage samples. For example, the four sequences obtained from the sewage sample obtained on 21st May 2014 (Fig. 1, black triangles) all grouped with different reference sequences. Multiple variants were also observed amongst the sequences from 7th May 2014 (dark grey triangles), 4th June 2014 (dark grey circles), 16th July 2014 (black rhombus), and 7th January 2015 (light grey circles). All 21 of the sequences obtained from the 26th March 2014 sample (black circles) grouped with the subtype 3c reference sequence, but these sequences nevertheless displayed considerable diversity, although differences based on the analysis of a short subgenomic region should not be overinterpreted. Since mixed infection with HEV in immunocompetent individuals is rare, although not undocumented [Smith et al., 2013], these observations imply that the HEV variants detected in sewage derive from multiple individuals.

HEV genotypes other than HEV‐3 were not detected, despite the presence of HEV‐1 in 11% of Scottish (Fig. 1) and 30% of English hepatitis patients [Ijaz et al., 2014]. The explanation for the failure to detect HEV‐1 in sewage may be that the number of individuals in the Scottish population infected with HEV‐3 is much higher than with HEV‐1. The incidence of HEV infection in England and Wales has been estimated at 60,000/year [Ijaz et al., 2014], which would suggest that there are about 6,000 seroconversions/year in Scotland. However, more than 98% of these infections are asymptomatic since screening of individuals with abnormal liver function tests or other signs of hepatitis only identified 93 HEV infections in Scotland during 2013 (http://www.documents.hps.scot.nhs.uk/giz/annual-report/hps-fsa-joint-annual-report-2013.pdf). In addition, while HEV‐3 infection appears to be an autochthonous zoonotic infection, most HEV‐1 infections in the UK are associated with recent travel to the Indian subcontinent where HEV‐1 infection is endemic [Ijaz et al., 2014]. Since there is no evidence for secondary transmission of HEV‐1 within the UK, the vast majority of HEV detected in sewage would be expected to derive from asymptomatic individuals infected with HEV‐3.

Faecal shedding of HEV does not persist for more than 4 weeks after the onset of symptoms [Aggarwal et al., 2000]. On the assumption that this period is similar for asymptomatic HEV‐3 infections, about 50 HEV‐infected individuals within the Seafield catchment area would be expected to be shedding virus in faeces at any one time (6,000 HEV seroconversions/year in Scotland would mean about 600/year in Edinburgh of whom 4/52 = 46 would be excreting virus in faeces at any one time). This estimate explains the high frequency with which HEV‐3 was detected in sewage samples and also why multiple lineages were detected within each sample. Even greater diversity would be observed if longer or more variable regions of the virus genome were analyzed or if variants present at low copy number were sampled by sequencing larger numbers of amplicons obtained at limiting dilution.

Most cases of human infection with HEV‐3 in Europe are thought to derive from the consumption of undercooked pig meat or pig products, although a direct linkage has been made in only a few instances. The finding that diverse strains of HEV‐3 can frequently be detected in untreated sewage (at a level of <2000 RNA copies per ml) means that appropriate precautions against infection should be taken by those working in sewage plants or in proximity to sewers. Further studies on the presence and diversity of HEV in sewage may provide insights into the epidemiology of this ubiquitous yet little‐understood virus.
